# Improving Osteosarcoma Treatment: Comparative Oncology in Action

**DOI:** 10.3390/life12122099

**Published:** 2022-12-14

**Authors:** Lidia Tarone, Katia Mareschi, Elisa Tirtei, Davide Giacobino, Mariateresa Camerino, Paolo Buracco, Emanuela Morello, Federica Cavallo, Federica Riccardo

**Affiliations:** 1 Molecular Biotechnology Center “Guido Tarone”, Department of Molecular Biotechnology and Health Sciences, University of Torino, Via Nizza 52, 10126 Torino, Italy; 2 Department of Public Health and Paediatrics, University of Torino, Piazza Polonia 94, 10126 Torino, Italy; 3 Stem Cell Transplantation and Cellular Therapy Laboratory, Paediatric Onco-Haematology Department, Regina Margherita Children’s Hospital, City of Health and Science of Torino, 10126 Torino, Italy; 4 Department of Veterinary Sciences, University of Torino, Largo Paolo Braccini 2, Grugliasco, 10095 Torino, Italy

**Keywords:** osteosarcoma, comparative oncology, immunotherapy, CSPG4

## Abstract

Osteosarcoma (OSA) is the most common pediatric malignant bone tumor. Although surgery together with neoadjuvant/adjuvant chemotherapy has improved survival for localized OSA, most patients develop recurrent/metastatic disease with a dismally poor outcome. Therapeutic options have not improved for these OSA patients in recent decades. As OSA is a rare and “orphan” tumor, with no distinct targetable driver antigens, the development of new efficient therapies is still an unmet and challenging clinical need. Appropriate animal models are therefore critical for advancement in the field. Despite the undoubted relevance of pre-clinical mouse models in cancer research, they present some intrinsic limitations that may be responsible for the low translational success of novel therapies from the pre-clinical setting to the clinic. From this context emerges the concept of comparative oncology, which has spurred the study of pet dogs as a uniquely valuable model of spontaneous OSA that develops in an immune-competent system with high biological and clinical similarities to corresponding human tumors, including in its metastatic behavior and resistance to conventional therapies. For these reasons, the translational power of studies conducted on OSA-bearing dogs has seen increasing recognition. The most recent and relevant veterinary investigations of novel combinatorial approaches, with a focus on immune-based strategies, that can most likely benefit both canine and human OSA patients have been summarized in this commentary.

## 1. Introduction

**Figure 1:**
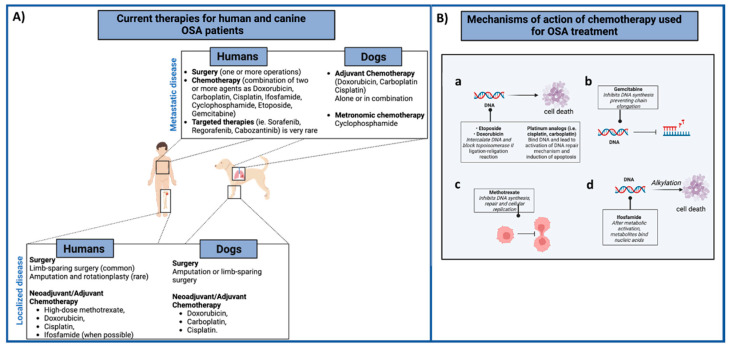
(**A**) Summary of the current therapies for human and canine OSA patients. In both species, the first-line treatment for the local tumor control (low panel) involves surgery with neoadjuvant/adjuvant administration of different chemotherapeutic agents, alone or combined. Metastatic OSA patients (high panel) are generally treated with surgery (metastasectomy) and chemotherapy regimens. (**B**) Mechanism of action of different chemotherapeutic agents (a–d) used for both human and canine OSA patients’ treatment are summarized. Images created with BioRender.com.

Osteosarcoma (OSA) is the most common primary malignant bone tumor affecting children and adolescents [1,2,3]. The standard of care for OSA treatment has not significantly changed since the 1980s, when Rosen et al. introduced pre-operative chemotherapy [1,3,4,5,6]; the combination of surgery plus neoadjuvant/adjuvant chemotherapy with high-dose methotrexate (HDMTX), doxorubicin (DOXO) and cisplatin (DDP), with the possible addition of ifosfamide (IFO), strikingly increased the long-term survival of OSA patients with localized disease from 10–15% to 65–70% [7,8,9]. Nevertheless, about 40% of patients will suffer from recurrences within a few years and prognosis becomes very poor in these cases, with the impact of second-line chemotherapy for recurrent OSA being much less well defined [3]. The clinical scenario is further complicated by the fact that the majority of patients have micro or already evident metastases at diagnosis [3,10]; the lungs comprise 90% of metastatic sites, with other extra-pulmonary distant metastases, mainly involving the bones, bone marrow and liver, to a lesser extent, accounting for a further 8–10% [3,11,12]. Conventional treatments mostly fail in these patients, and the 5-year survival for metastatic OSA drops below 20%, meaning that this tumor is still a critical pediatric oncological issue [13,14] (Figure 1). The identification of innovative and efficient therapeutic approaches to improve OSA survival rates and reduce the toxicity associated with conventional chemotherapies is a significant and currently unmet need. Indeed, standard chemotherapeutic protocols have considerable acute and late side effects, including leukopenia, liver and kidney functional impairment, neurotoxicity, gastrointestinal reactions and cardiomyopathy, among others, that can be severe and potentially permanent [14,15,16]. This is far from acceptable considering the very young age of OSA patients.

However, the identification of novel treatments remains a challenge in the oncology panorama; the scarce knowledge of OSA biology and lack of common targetable antigens mean that no real therapeutic options have been proposed for decades [17].

**Figure 2:**
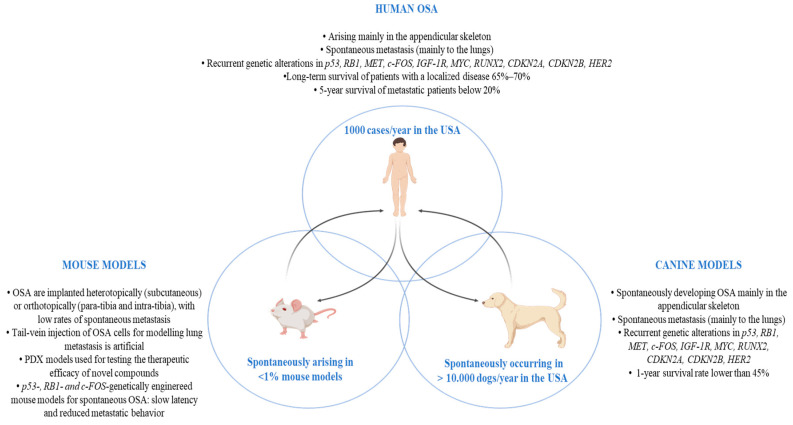
Schematic summary of the main OSA features in pre-clinical mouse and canine models and in humans. Abbreviations: OSA (osteosarcoma); p53 (Tumor protein p53); RB1 (retinoblastoma 1 gene); MET (MET Proto-Oncogene, Receptor Tyrosine Kinase); c-Fos (Fos Proto-Oncogene, AP-1 Transcription Factor Subunit); IGF-1R (insulin-like growth factor receptor-1); MYC (MYC Proto-Oncogene, BHLH Transcription Factor); RUNX2 (RUNX Family Transcription Factor 2); CDKN2A (Cyclin-Dependent Kinase Inhibitor 2A); CDKN2B (Cyclin-Dependent Kinase Inhibitor 2B); HER2 (Human epidermal growth factor receptor 2); PDX (patient-derived tumor xenograft).

In order to foster the identification of new treatments in OSA, it is necessary to identify and develop more reliable pre-clinical models that better mimic OSA tumor heterogeneity and its microenvironment. Mouse models have been of undoubted relevance in in-vivo OSA research (Figure 2), in addition to in vitro approaches that include the use of established cell lines. However, it is worth noting that spontaneous OSA is very rare in mice, and, as a consequence, the study of OSA tumor growth and metastasization mostly relies on either the implantation of murine OSA cell lines into syngeneic animals, or that of either human OSA cells (xenograft) or patient-derived tumors (PDX) into immunocompromised mice [18,19,20]. In 2013, the Pediatric Preclinical Testing Program (PPTP) established that PDX can act as efficient pre-clinical models for the testing of novel therapeutic agents for OSA treatment [21], as they can be considered as “avatars behind the scenes”. These models might facilitate the development of comparative trials in “real time” by treating mice implanted with derived tumors using the same therapeutics as the patient and comparing tumor response. The main advantage of this approach is the possibility of evaluating, in the PDX, novel potential treatment combinations and their side effects in addition to testing the therapy chosen for the human patient, thus contributing to improving the precision therapy concept in humans [22].

In general, the most frequent route of tumor cell challenge is subcutaneous injection, although this has the major limitation of not being able to fully recapitulate the tumor microenvironment, which is a highly significant factor in tumor growth, progression and mainly for chemoresistance in OSA [20,23,24]. While intravenous injection is a quicker model for the study of lung metastases, it does not mimic the steps that cells must be able to complete (i.e., invasion, intra- and extravasation) prior to giving rise to new lesions in distant organs, making this a somewhat artificial model. Para- and intratibial cell injection allows OSA to initiate and expand into the “accurate” site, to establish contact with the proper microenvironment and to have access to the vasculature, thus metastasizing to distant sites, including the lungs. In this way, the nature of human OSA growth and spontaneous metastasization is better recapitulated [20,25,26]. Nevertheless, intraosseous growth makes tumor monitoring difficult.

Advances in the characterization of the molecular bases of different cancer types and the development of sophisticated techniques to manipulate the mouse genome have led to the development of several transgenic mice that spontaneously develop OSA. Specifically, animals that carry deletion in the tumor protein p53 (p53) [27,28,29,30] and/or retinoblastoma 1 (RB1) genes [27,29,30] and others that over-express the Fos Proto-Oncogene, AP-1 Transcription Factor Subunit (c-FOS) [31,32] in primitive mesenchymal cells or osteoblasts have been generated and bear different features regarding tumor latency, penetrance and the development of spontaneous metastasis (reviewed in [20]). However, the average tumor latency in these mice is very slow, with the development of OSA at approximately 1–2 years of age, and reduced metastatic behavior compared to human OSA. In addition, other diseases caused by this genomic manipulation might develop concomitantly in some cases [33].

Therefore, although mice provide an important pre-clinical model for the investigation of specific molecular processes in the initiation and development of OSA, they cannot faithfully recapitulate some of the important features of human OSA, including long-term interactions with the immune system [24,34]. Moreover, murine models possess intrinsic limitations that reduce their power of translation into a clinical and therapeutic perspective. Indeed, mice have lower tumor mutational burden, a higher tolerance to drugs than humans, bone marrow that is less sensitive to cytotoxic agents and radiotherapy and limited “inter-patient” heterogeneity [20,24,34].

Together with the need to identify complementary methods to strengthen the successful translation of cancer research “from bench to bedside” comes the search for alternative models, and this search brings dogs affected by spontaneous tumors into the limelight of the comparative oncology stage.

In this commentary, we will summarize the most recent and relevant studies that signal OSA-bearing dogs as being appropriate models for the evaluation of emerging therapies in a way that can have a significant clinical impact on immuno-oncology research to the benefit of both species (Table 1).

## 2. Comparative Oncology

The origin of the “One Medicine” concept dates back to the 19th century [63]. The aim was to promote collaboration between the medical and veterinary disciplines through the study of naturally occurring spontaneous diseases that are comparable to each other to the benefit of both humans and animals. While principal interest was initially directed towards infectious diseases, mainly zoonosis, “One Medicine” then evolved into “One Health”, which is a more complex initiative that aims to stimulate a multidisciplinary approach towards health promotion, rather than solely the treatment of disease, encompassing the health of humans, animals and the environment. Both of these concepts rely on the existence of high similarity in physiology, anatomy, exposure and environmental influences on diseases across species, and hold the intent of improving the understanding and resolution of both human and animal health problems. Starting with these ideas, specific focus has been placed on cancer, leading to the conceptualization of the comparative oncology branch. By definition, it aims to integrate “the study of naturally occurring cancers in veterinary patients into more general studies of cancer biology and therapy”, to the benefit of both human and animal oncological research. Indeed, spontaneous tumors in animals, especially in dogs, have similar biology, genetic background, pathophysiology, risk exposure and clinical behavior, therefore more closely mirroring human cancer [34,64,65,66]. The parallel study of cancer in humans and dogs can enhance our understanding of the factors that lead to tumor formation, progression and the failure of therapy.

On the back of this drive, the National Cancer Institute’s Center for Cancer Research (CCR) promoted the Comparative Oncology Program (COP), in 2003, with the goal of focusing on the use of naturally occurring cancer in dogs as a model for human tumors [65]. Some years later, a European project also joined this concept, launching the LUPA project with a similar purpose [67]. Lastly, the Canine Comparative Oncology and Genomics Consortium established a biospecimen repository as a resource to facilitate comparative genomics and the identification of tumor targets that are relevant for both species.

From that moment on, the importance of comparative oncology spread all over the scientific world to the point that veterinary trials in pet animals have been introduced as a relevant step in the workflow of the clinical development of novel anticancer therapies [65,68,69]. The numerous published studies conducted in the veterinary setting have highlighted the importance of comparative oncology in advancing key questions in the biology of cancer and response to therapy that cannot be sufficiently resolved using traditional mouse models. Dogs are an immunologically outbred population that develop tumors spontaneously, with slow and stepwise progression, in the context of an intact immune system that shapes the tumor microenvironment. These features are important when evaluating immunotherapy responses. Moreover, in the immunotherapy field, it is important to consider that the immune systems of humans and dogs show greater similarities than those of humans and mice.

## 3. OSA in Pet Dogs

In the panorama of comparative oncology, OSA is one of the best-characterized spontaneous canine malignancies with a high translational relevance to the human clinical context (Figure 2). OSA occurs in 10,000 dogs/year in the USA [70,71,72], with a wider peak of incidence being observed in larger and giant breed dogs [73]. Canine OSA most commonly occurs in the appendicular skeleton, and usually affects the metaphysis of long bones, such as the distal radius, proximal humerus, distal femur, proximal and distal tibia and ulna [74]. Numerous investigations have highlighted the pathological, biological and clinical similarities between human and canine OSA [34,75,76,77,78].

The genomic characterization of OSA in pet dogs has recently shed light on the molecular alterations shared by human and canine OSA, including the overall mutational burden; the high frequency of p53 mutations; copy-number aberrations of several key genes, such as RB1, MET Proto-Oncogene, Receptor Tyrosine Kinase (MET), c-FOS, insulin-like growth factor receptor-1 (IGF-1R), MYC Proto-Oncogene, BHLH Transcription Factor (MYC), RUNX Family Transcription Factor 2 (RUNX2), Cyclin-Dependent Kinase Inhibitor 2A (CDKN2A), Cyclin-Dependent Kinase Inhibitor 2B (CDKN2B), Human epidermal growth factor receptor 2 (HER2) and others; and pathway dysregulations, such as in ERK and PI3K–mTOR [79,80,81]. These common alterations lead to similar behavior in human and canine OSA, such as tumor progression, immune evasion and a high propensity to give rise to recurrences and metastases. The standard-of-care treatment for canine patients includes limb amputation/-sparing surgery followed by either systemic adjuvant platinum-based or doxorubicin chemotherapy, used alone or in combination. Such treatments are only modestly effective in prolonging the survival of OSA-affected dogs, with a 1-year survival rate of below 45% [82]. Moreover, in most cases, metastases occur rapidly and spread to secondary organs, most frequently to the lungs, although other sites including distant bone, liver, spleen and lymph nodes have been described [83], dramatically impacting the survival rate [73,82] (Figure 1).

## 4. Translational Studies of Combinatorial Chemotherapeutic Approaches

Both human and canine OSA present an unsolved oncological issue; both are chemo- and radio-resistant in their advanced stages, while intra- and interpatient tumor heterogeneity in both hamper the development of targeted therapies that can be broadly applied. Specifically, an additional comparative advantage for OSA can be found in the fact that it is rare in the pediatric population, while its incidence in dogs is approximately 27-fold higher [82]. Considering the young age of human OSA patients and the low number of new cases/year, it is difficult to effectively recruit patients for highly statistically powerful trials that can test novel therapies. Therefore, veterinary trials in dogs possess the potential to provide an appealing “platform” for investigations into treatments with high informative and translatable value for the human clinical context.

### 4.1. In Vitro Canine OSA Models

The possibility of proving the efficacy of novel combinatorial approaches in pet dogs has become so appealing that even in vitro studies often include canine cell lines along with human cells, thus acting as a preliminary basis for proposals of in-dog testing to accelerate in-human trials. For example, Chirio et al. recently demonstrated the potential of using DOXO-loaded nanoparticles for the treatment of both human and canine OSA in vitro [35]. This proof of concept may promote the clinical evaluation of this strategy in a comparative trial that aims to overcome multidrug resistance to chemotherapy. Moreover, Yang et al. recently utilized both human and canine OSA cells, in vitro, to test the combination of Sorafenib, a multi-tyrosine kinase receptor inhibitor (TKRi), and conventional chemotherapeutic agents, including DOXO [40]. Actually, the molecule Sorafenib is not new to human clinics, as it has previously been tested in several trials with quite positive results [84,85,86]. A recent study demonstrated that DOXO administration is able to upregulate the expression of the checkpoint molecule PD-L1 on human OSA cells, consequently causing an immunosuppressive microenvironment and treatment failure. Administration with Sorafenib could revert this event, inhibiting DOXO-induced PD-L1 upregulation and inducing an increase in the cytotoxic CD8^+^ T cells infiltrating the tumor [87]. This work strongly supports the positive benefits of the Sorafenib/DOXO combination. In this way, the results of Yang et al. show that the use of Sorafenib in combination with DOXO provides a synergistic effect in the treatment of human and canine OSA cells, laying the foundation for evaluating this strategy in a comparative oncology trial before in-human translation [40].

Another approach to overcoming chemoresistance to platinum-based therapies has been proposed by Inkol and collaborators [41], again in an in vitro comparative study. Since platinum agents are shuttled within cells via copper transporters, the impact on chemotherapy response when these pathways are inhibited was evaluated in both human and canine OSA cells. In both cases, transporter inhibition was effective in sensitizing OSA cells and, therefore, in reducing acquired chemotherapy resistance, laying the foundation for a comparative veterinary trial.

### 4.2. In Vivo Canine OSA Models

Several modifications to the standard treatment protocols have been explored in human clinical trials without a clear benefit for patients with advanced or relapsed/refractory OSA being obtained, with this being in part due to difficulties in recruiting a sufficiently high number of patients to collect statistically significant safety and efficacy data. Consequently, some trials have been conducted using dogs as a model for advanced OSA.

Recent examples with potential translational value include the use of Auranofin, which is a compound that can inhibit an enzyme named TrxR2 that is probably involved in driving OSA lung metastasis through the regulation of reactive oxygen species in the mitochondria. Auranofin is already used for other purposes, including rheumatoid arthritis, in both humans and dogs, and the authors have proposed an interesting reuse of this product in its potential clinical application against OSA. In canine patients, Auranofin has been demonstrated to improve overall survival in combination with conventional chemotherapy, delaying the development of pulmonary metastasis [46].

A great deal of attention has also been directed towards the use of TKRi in combination with standard of care. One of the most relevant TKRi in veterinary clinics is toceranib phosphate (Palladia), which is a multi-target TKRi that acts against vascular endothelial growth factor receptor (VEGFR), platelet-derived growth factor receptors (PDGFR), c-Kit, colony-stimulating factor 1 receptor (CSF1R) and fms-like tyrosine kinase (FLT3), which are commonly altered in many cancers, including OSA [88]. It has been suggested that the use of toceranib phosphate, in combination with chemotherapy, may be effective in treating canine metastatic OSA with high PDGFR, VEGFR2 and c-Kit expression [42,43]. From the translational point of view, toceranib is an analogue of sunitinib, which is an FDA-approved drug for the treatment of other cancers [89] that has shown promising effects in human OSA cell lines in vitro and in xenograft mouse models [36], although no clinical data are available to date. Veterinary trials in OSA canine patients may therefore be informative in proposing the evaluation of toceranib as a combinatorial drug to increase chemotherapy efficacy in the presence of metastasis to the benefit of both humans and animals with OSA.

The use of bisphosphonate therapy, including zoledronic acid and pamidronate, in association with standard of care for the treatment of OSA has been investigated in several in vitro studies as well as in animal OSA models, including dogs [44,45,90,91,92,93]. While their effectiveness in the management of primary disease is unclear, more appealing evidence has been reported for the management of metastases, which is an important achievement considering the aggressiveness of OSA metastatic spread.

The use of gemcitabine has been proposed, in recent years, for the treatment of pulmonary metastases. Gemcitabine is a deoxycytidine analogue that is already used for the management of hematological and human solid tumors, including relapsed and unresectable high-grade OSA [94,95,96,97,98]. Rodriguez and collaborators proposed the administration of gemcitabine, via the aerosol route, in canine OSA patients, and demonstrated the potential that this approach possesses in improving drug delivery to the lungs without collateral effects; in addition to its high tolerability, this strategy was observed to be able to control lung metastases [37]. On the basis of these positive results in dogs, a human clinical trial recently started [38].

New formulations and new administration systems have also been proposed for the use of paclitaxel in dogs with high-risk invasive tumors, potentially including advanced OSA, with interesting data for the transfer of knowledge from the “bench” to the “bedside” being provided and new therapeutic approaches in humans being suggested [99,100,101]. For example, in vitro pre-clinical and clinical data on the use of microfragmented adipose tissue as a delivery system for paclitaxel in canine mesothelioma [102,103] were fundamental to the Phase I authorization of adipose-derived mesenchymal stem cells loaded with paclitaxel (PacliMES) (EudraCT No. 2020-005928-11 [104]). This is another new encouraging therapeutic approach that could be investigated in the OSA setting.

These recent studies, which are summarized in Table 1, are just a few examples of the multiple attempts to improve the conventional chemotherapeutic protocols for OSA, especially for the treatment of advanced disease.

Another topic that deserves mention is the search for combinatorial approaches using phytopharmaceuticals, plant-derived compounds that are acquiring increasing importance as complementary treatments for different types of cancer, including OSA [105]. Indeed, phytochemicals and some derivatives present in plants possess intrinsically low toxicity in normal cells, but may be able to mediate several anticancer effects, including increasing antioxidant status, inhibiting proliferation, inducing cell cycle arrest and apoptosis and regulating the immune system [106].

Therefore, the anticancer and chemo-adjuvant properties of phytochemicals have been tested in pre-clinical and clinical trials [106,107], including the OSA setting, in recent years.

Of these phytochemicals, curcumin, [(1E,6E)-1,7-bis (4-hydroxy-3-methoxyphenyl)-1,6-heptadiene-3,5-dione or diferuloylmethane], a phenolic compound obtained from the turmeric plant *Curcuma longa* L., has been widely investigated for its anti-inflammatory, antioxidant and chemo-preventive properties [108,109]. In particular, several in vitro studies have demonstrated the potential of treatment with curcumin in inhibiting both human and canine OSA cell proliferation and metastatic potential, inducing an apoptotic effect [110]. However, poor cellular uptake, low solubility and low bioavailability have limited the successful application of curcumin in vivo [111]. Therefore, a number of different strategies to overcome these limitations have been explored, including the use of nanostructures, such as liposomes and soy protein nanoparticles, and synthetically synthesized curcuminoid analogs [112,113,114]. The data demonstrate the high antitumor potential of these alternative formulations when used alone or in combination with chemotherapy, with promising results also being obtained in a small pilot study in canine patients affected by pulmonary metastasis, including OSA-derived ones, in which few patients displayed stable disease [112]. Moreover, it has been demonstrated that curcumin can synergize with chemotherapeutics and also reverse chemotherapy resistance, while favoring the protection of normal tissues from both DOXO and cisplatin toxicity thanks to its antioxidant effects [108,115]. In addition, curcumin can recover the bone defects caused by tumor erosion and surgery, and this could be important in supporting conventional OSA treatment [109].

Another compound found in several plants that has been widely investigated is trans-3,4′,5-trihydroxystilbene, best known as resveratrol (RSV). RSV is acquiring importance in the OSA field as, in addition to its anti-inflammatory and antioxidant effects and anticancer activity, which induces cell cycle arrest, apoptosis and autophagy, it promotes the formation and regeneration of bone and cartilage with protective properties for the viability and proliferation of bone marrow mesenchymal stem cells, thus favoring osteogenic differentiation [116].

Moreover, RSV can strengthen the cytotoxic effects of both DOXO and cisplatin on human OSA cells [105,117,118]. Interestingly, RSV has been demonstrated to suppress the activation of cancer cells with a stem phenotype [119,120], which are considered to be responsible for chemoresistance, thus further supporting the potential beneficial role of RSV treatment in association with chemotherapy.

The antitumor potential of RSV has also been proven in a veterinary setting against hemangiosarcoma cell lines [121]. Taken as a whole, these results also open up possible further investigations into the adjuvant administration of RSV for the treatment of canine OSA.

Other compounds, including saffron, have been tested against both human and canine OSA cells, alone and in combination with chemotherapeutics [122,123,124,125], all with the common final aim of enhancing therapeutic efficiency, diminishing drug use and reducing the phenomena of chemoresistance and related side effects.

Other interesting approaches include immune-based techniques, which deserve special attention. Indeed, immunotherapy is now considered a modern pillar of cancer care.

## 5. Translational Studies of Immune-Based Approaches

The potential role of the immune system in delaying the progression of OSA, thus prolonging survival in both humans and dogs, derives from the observation of outcomes when prosthetic reconstructions become infected [126,127,128]. As previously mentioned, OSA in pet animals arises spontaneously in an intact immune system, reproducing the interactions between the developing tumor and immune cells, as in human patients. Therefore, pet OSA patients are reliable models for the identification of novel and effective immune-based strategies and could be instrumental for the long-lasting cure of this disease. Moreover, as the use of checkpoint inhibitors (CIs) alone has failed to provide real benefits for the treatment of OSA in human clinical trials [129,130,131], dogs offer a relevant model for the testing of combinatorial approaches to unveil the most effective strategy to exploit the potential of CIs, also in the OSA setting. Dogs are certainly more predictive models for CIs than mice. An example of this can be found in the expression of the second ligand of PD-1, denoted as PD-L2, which is acquiring growing importance as a predictor of response to CIs in human patients, including OSA patients [132,133]. It binds to PD-1 with two- to sixfold higher affinity than PD-L1. It can be expressed by immune, stromal and cancer cells in humans, while its expression on mouse tumor cells is almost nonexistent. In addition, PD-L2 can have T-cell co-stimulatory functions in mice because it also binds to the repulsive guidance molecule b (RGMb), which is expressed on the surface of naive mouse T cells, macrophages, neutrophils and dendritic cells. These discrepancies between mice and humans contributed to making PD-L2 a neglected target [134]. However, in dogs, PD-L2 appears to have a dedicated co-inhibitory function as it does in humans, and its expression is a common feature of canine cancer cells [135], making dogs reliable models to study the effects of CIs and of immunotherapy in general.

A summary of key recent in-dog immunotherapy studies is presented below and summarized in Table 1.

### 5.1. Branded Targets

Human epidermal growth factor receptor 2 (HER2) is one of the potential OSA-associated antigens in both the veterinary and human settings. HER2 is overexpressed in about 60% and 40% of human and canine primary OSA, respectively. Its overexpression correlates with higher metastatic rates, chemo-resistance and shorter survival [136,137].

Different strategies for targeting HER2 have been developed and pre-clinically/clinically tested in OSA models. Following successful pre-clinical studies in murine models of OSA [47,138], which demonstrated the ability of HER2-targeted CAR-T cell therapy to kill OSA cells and derived metastases, this strategy has also been evaluated in human patients affected by recurrent OSA. Despite initial positive clinical evidence [139], safety concerns were raised by Morgan and co-authors [48] as several patients treated with CAR-T cell infusions developed cytokine storms, which were not predicted by preclinical studies conducted in murine models. To solve the issue, Mata et al. turned to dogs and developed second-generation canine HER2-targeted CAR-T cells. The ability of these cells to recognize and kill both human and canine HER2+ target cells in an antigen-dependent manner, with similar cytolytic activity to the human HER2-targeted CAR-T cells, was demonstrated, but cytokine production was lower [49]. This paves the way for their evaluation in large animals before the development of future clinical trials in humans.

A different approach to targeting the HER2 antigen was designed in 2016; a recombinant Listeria monocytogenes (Lm)-based vaccine expressing a chimeric human HER2 fusion protein was tested in a Phase I veterinary clinical trial [50]. The vaccination strategy, in an adjuvant setting, was found to be effective in impairing the development of lung metastasis and in prolonging the overall survival of OSA-affected dogs. On the basis of these positive results, a lyophilized formulation of the live Listeria vector vaccine (the canine OSA vaccine, live Listeria vector; COV-LLV) received a conditional license from the United States Department of Agriculture (USDA) in 2017 for the adjuvant treatment of dogs with OSA [50,140]. Importantly for the comparative oncology field, the positive results obtained in canine patients led to a Phase Ib clinical trial utilizing this vaccine, named ADXS31-164, in adult patients with HER2-expressing tumors (NCT02386501), and it has now been licensed for development in the pediatric OSA setting by OS Therapies in conjunction with the NCI Children’s Oncology Group [141]. Unfortunately, a larger veterinary study raised safety concerns, including the occurrence of significant adverse effects, such as Listeria abscess and severe infections following administration. The potential hazard of the zoonotic spread of the disease in humans led to the vaccine license for dogs being terminated by the company [142,143]. Nevertheless, these results highlight the potential clinical impact of targeting HER2 for the treatment of OSA in a context of minimal residual disease.

A different approach to targeting HER2 in OSA via cancer vaccination has recently been suggested by Doyle and collaborators [51] in another Phase I/II veterinary study. In this trial, the authors identified homologous domains shared by EGFR, HER2 and HER3, and tested the immunogenicity and the clinical potential of EGFR/HER2 specific peptide-based vaccination in dogs with ErbB/Her2-overexpressing tumors, including OSA. The results show that an antibody response was induced with relevant biological activity that inhibited ErbB/HER2 signaling, in both canine and human tumor cells, and tumor growth in vitro. Clinically, this vaccination strategy was found to be effective in a first group of OSA-immunized dogs [51].

In the search for other targetable oncogenes in OSA, several studies have also focused on the role of insulin-like growth factor-1 (IGF-1) and its receptor (IGF-1R), since IGF-1R has been found to be overexpressed in both human and canine OSA, as well as being correlated with a malignant phenotype [144]. However, its targeting gave poor results in a randomized controlled trial in OSA-bearing dogs and in human OSA patients [52,53]. Renewed interest in the IGF-1 pathway has nevertheless been generated by the observation that the second receptor for IGF-1 (IGF-2R) is also expressed in both human and canine OSA tissues, indicating that it may have potential as a comparative therapeutic target [145,146]. Recent studies that exploit monoclonal antibodies targeting IGF-2R, conjugated to various different cytotoxic radioisotopes, in pre-clinical canine and in vitro human models have suggested that this radio-immunotherapy strategy is able to suppress tumor growth [145,147]. Although these studies are in their infancy, they are laying important foundations for new possibilities in OSA treatment.

### 5.2. Other Immunomodulatory Strategies

A different immunization strategy has recently been reported in a veterinary trial that combines standard of care (surgery and chemotherapy) and a peptide-based anticancer vaccine in dogs with non-metastatic OSA. In this study, the vaccine was composed of non-conventional endoplasmic reticulum stress response-derived immunogenic peptides (ERstrePs) that are released following the infection of OSA cells with Salmonella. The secretome, composed of non-mutated tumor antigens that are expressed by ER-stressed tumor cells, but not by healthy, non-ER stressed cells, was used to adjuvantly vaccinate OSA-bearing dogs. The dermal administration of this peptide-based vaccine was well tolerated, and antitumor efficacy was reported, with the time to metastasis and tumor-specific survival being longer in vaccinated dogs than in historical controls. The induction of an antitumor humoral and cellular immune response was also observed [54,55].

In a preliminary proof-of-concept study, Magee et al. [148] started to investigate the potential of a tri-modal immuno-radiotherapy approach that combines in situ radiation therapy and an intratumor injection of the immunocytokine fusion protein hu14.18-IL2, which is made up of human recombinant IL2 fused to the humanized antidisialoganglioside (GD2) monoclonal antibody (mAb). The resulting in situ immune-radiotherapy strategy was applied to companion dogs affected by advanced metastatic tumors, including OSA. Because of this treatment, however, non-irradiated metastatic lesions may become a niche for immunosuppressive cells, leading to systemic immune tolerance that can limit the benefits of in situ application. The authors added systemic targeted radionuclide therapy to the approach to avoid this issue by abrogating immunosuppression in secondary lesions, modulating the tumor microenvironment in order to promote the propagation of an antitumor immune response to multifocal metastatic disease, with promising results in dogs [148]. Indeed, the immunomodulation of tumor-infiltrating lymphocytes was observed after treatment, with an increase in CD45^+^, B, CD8^+^ T and highly cytolytic NK cells, although an increase in T regulatory cells was also detected [148].

The most appealing evidence on immune modulation in OSA can currently be found in the use of the liposome-encapsulated lipophilic derivative of muramyl dipeptide (L-MTP-PE). This immunomodulatory agent can stimulate a systemic anticancer immune response via the activation of macrophages and monocytes [149,150]. The activation of these immune mediators may lead to tumor cell elimination via both direct lysis and the release of tumoricidal pro-inflammatory cytokines. The adjuvant administration of L-MTP-PE has proven to be a highly effective treatment for canine OSA in a randomized veterinary clinical trial [57,151]. When combined with chemotherapy, L-MTP-PE was found to be more effective in counteracting metastatic spread and improving dog survival than placebo-treated controls, as it was able to enhance both monocyte activation and the cytotoxic activity of macrophages against OSA cells [56,57,58,59]. The positive results obtained in veterinary medicine have promoted the development of L-MTP-PE in human clinical trials, with Food and Drug Administration (FDA) approval for the adjuvant treatment of non-metastatic human OSA patients being achieved [149,152,153,154,155].

This success suggests that immunotherapeutic approaches that target the innate immune system still hold promise for OSA patients. In a further step in this direction, Regan and collaborators have proposed another combinatorial strategy for the treatment of OSA-derived lung metastasis, with a comparative goal. Pre-clinical studies in murine models of metastatic cancers have suggested that inflammatory monocytes that express the chemokine receptor CCR2 are preferentially attracted, by the production of CCL2, to early metastatic sites where they can differentiate into metastasis-associated macrophages (MAMs), promoting metastatic colonization. Regan and co-authors have demonstrated that losartan, normally used as an angiotensin blocker for the treatment of hypertension, is effective in impairing the CCR2/CCL2 axis, preventing monocyte recruitment and stimulating an antimetastatic effect in preclinical mouse-models [156]. The group then proposed this strategy in a veterinary trial after positive results in mice. In this recent study, the authors combined monocyte-targeted immunotherapy with losartan and toceranib phosphate (Palladia), which has already been approved in veterinary medicine and is a functional homologue of sunitinib, the multi-kinase inhibitor approved for investigational use in human OSA. In this trial, which enrolled dogs with advanced lung metastatic OSA, the combinatorial approach was observed to be safe with a clinical benefit rate of 50%. These promising results support the evaluation of this approach as a novel strategy for the treatment of high-risk metastatic OSA patients, both in the human and veterinary settings [61].

In the same year, a first-in-dog Phase I clinical trial indicated that recombinant human IL-15 has potential in the treatment of OSA-derived lung metastasis. There is emerging interest, in the immune-oncology field, in the development of strategies to activate immune cell subsets besides T cells, such as natural killer (NK) cells, that might play a relevant role in cancer elimination. IL-15 has therefore been selected because of its ability to stimulate powerful antitumor immune responses via the activation of endogenous cytotoxic NK cells. However, human clinical trials using IL-15 monotherapy for patients with advanced cancer have frequently failed, and are often limited by the onset of systemic toxicity [157,158]. Rebhun et al. exploited dogs with spontaneous advanced OSA as a suitable model with which to test the safety profile and clinical activity of inhaled rhIL-15. The positive results of this study, which demonstrated the induction of a cytotoxic immune response that correlated with a clinical benefit, further support the investigation of inhaled rhIL-15 in the treatment of dogs with metastatic OSA, with the final comparative aim of the safer application of this treatment in human OSA patients [62].

## 6. Chondroitin Sulfate Proteoglycan 4 (CSPG4): A Novel OSA Immunotherapeutic Target

The search for innovative therapeutic targets in OSA is ongoing, and CSPG4 is emerging as an appealing molecule. It is a transmembrane protein that is either expressed on the cell surface as an N-linked glycoprotein of ~250 kDa, or as a ~450 kDa N-linked glycoprotein associated with a proteoglycan component.

Physiologically, CSPG4 retains limited expression in healthy adult tissues, mainly in a restricted population of partially differentiated progenitor cells, including mesenchymal stem cells, the microglia in the central nervous system and vascular pericytes [159,160,161,162,163,164]. However, it is overexpressed in several hematological and solid tumors, including melanoma, oligodendrocytomas, gliomas, renal cell carcinomas, chondrosarcomas and pancreatic and triple-negative breast carcinomas. Functionally, thanks to its extended extracellular domain, CSPG4 may bind several growth factors, such as basic fibroblast growth factor (bFGF) and PDGF, and present them to their RTK, potentiating and sustaining the activation of these pathways. Alternatively, it may bind to metalloproteinases and collagens in the extracellular matrix, playing an important role in the communication between the external and internal compartments of tumor cells. Its overexpression has therefore been linked to the regulation of several cancer-related pathways, including the support of a highly proliferative, migratory and invasive phenotype of tumor cells and chemo- and radio-resistance. CSPG4 has been shown to be directly involved in aggressive tumor behavior in several cancer histotypes, as it is also overexpressed on cancer stem cells and is associated with poor prognosis [76,77,160,161,162,164,165,166].

In 2016, Sato and collaborators suggested that CSPG4-overexpressing progenitor cells in murine models, in that case, pericytes, may be potential sarcoma- and OSA-initiating cells [164]. Moreover, a first report on the possibility of targeting CSPG4 in OSA was suggested by in vitro experiments using anti-CSPG4 CAR-T cells [167]. Cytokine-induced killer lymphocytes (CIK), engineered with a CAR that was directed against CSPG4, have more recently shown effectiveness in eliminating many types of soft-tissue sarcoma-derived cells, both in vitro and in vivo in immunodeficient mice [168]. Nevertheless, few data are available on CSPG4 expression and function in OSA.

Considering the appealing features of CSPG4 as an immunotherapeutic target, we have recently focused our attention on the expression and functional role of this oncoantigen in human and canine OSA. We have demonstrated that CSPG4 is overexpressed on human and canine OSA biopsies and cell lines, and on the derived CSC-enriched osteospheres. Moreover, our data suggest that there is a correlation between poor prognosis and high CSPG4 mRNA and protein levels in human and canine OSA patients [163]. Preliminary in vitro studies have signaled the potential efficacy of CSPG4 immune-targeting, with anti-CSPG4 antibodies that impair OSA cell proliferation, migration and sphere formation, when used alone and in combination with DOXO treatment [165]. Based on these results, and on the safety, immunogenicity and potential clinical benefit of a chimeric human/dog (HuDo)-CSPG4 DNA vaccine that we recently tested in the adjuvant setting in dogs affected by spontaneous CSPG4-positive oral melanoma [166], we are now also evaluating HuDo-CSPG4 DNA vaccination in OSA canine patients. A veterinary trial is ongoing (Tarone et al., manuscript in preparation), providing fresh hope for the development of a new therapeutic option that can be successfully applied in both veterinary and human OSA management.

## 7. Conclusions

OSA is still one of the most challenging oncological issues to face, and therapeutic options have not changed over the past 30 years, resulting in a dismally poor prognosis for metastatic and relapsed/refractory patients.

There is a considerable need for more useful pre-clinical models if advances in OSA clinical treatment are to be successfully made, and, together with a new awareness of the significant similarities between human and canine OSA at various levels, including molecular genetic alterations as well as biological and clinical behavior, this need has driven the use of OSA-bearing dogs as a highly predictive and translational model for human OSA (Figure 1 and Figure 2). Especially in the era of cancer immunotherapy, spontaneous canine tumors that develop in a mutually shaping immune-competent microenvironment represent a major opportunity for effectively testing the antitumor and antimetastatic potential of novel immune-based strategies. The ultimate goal here is to increase our knowledge of OSA biology and of therapeutic opportunities that can benefit both dogs and humans.

The importance of comparative oncology has also been proven by efforts to integrate pet dogs affected by spontaneous tumors, including OSA, into the development paths for new anticancer therapies. In this way, veterinary patients may have access to novel and promising therapeutic approaches while contributing to the acquisition of more robust and relevant data that can be used to better design human clinical trials and reduce translational failure.

## Figures and Tables

**Table 1:** Overview of the recent translational studies of combinatorial chemotherapeutic and targeted therapies and immunotherapeutic strategies.

	Treatment	Testing	Results	Authors
**Single treatments (chemotherapy or targeted therapies)**	**Doxorubicin-loaded nanoparticles**	Human and dog cell lines, in vitro	Increased drug uptake and cytotoxicity in chemo resistant cell lines	Chirio et al., 2022 [35]
	**Sunitinib (TKI)**	Human cell lines in vitro Human PDX models in vivo	Reduced tumour burden, microvessel density and suppression of pulmonary metastasis	Ram et al., 2015 [36]
	**Gemcitabine**	Pivotal study in metastatic OSA-bearing dogs Phase I clinical trial	Aerosol administration of Gemcitabine, control of lung metastasis Treatment of human patients with solid tumors and lung metastasis (including OSA)	Rodriguez et al., 2010 [37]; Gordon et al., 2020 [38]; *Ongoing* https://clinicaltrials.gov/ct2/show/NCT03093909, (accessed on 13 December 2022) [39]
**Combination therapies** **(chemotherapy + targeted therapies/other compounds)**	**Doxorubicin +** **Sorafenib (multi-kinase inhibitor)**	Human and dog cell lines, in vitro	Synergistic effects on tumor cell proliferation	Yang et al., 2022 [40]
	**Platinum-based chemotherapy + copper transporter inhibitors**	Human and dog cell lines, in vitro	Decreased tumor cell proliferation, migration and clonogenic potential, increased apoptosis	Inkol et al., 2020 [41]
	**Carboplatin+ Toracenib phosphate (TKRi targeting VEGFR, PDGFR; CSF1R; FLT3)**	Canine OSA cell lines, in vitro Canine PDX models, in vivo Prospective phase I veterinary trial	Decreased tumor cells growth, migration and invasion, in vitro Reduced tumor size in vivo 2 OSA-bearing dogs enrolled, experienced progressive disease	Sanchez-Céspedez et al., 2020 [42]; Wouda et al., 2018 [43]
	**Zoledronic acid and pamidronate alone or combined with chemotherapy**	Orthotopic canine PDX model Retrospective study	Inhibition of osteolysis following engraftment and decreased metastasis Decreased bone pain after chemotherapy	Wolfe et al., 2011 [44]; Lim et al., 2016 [45]
	**Carboplatin+ Auronafin**	Pilot veterinary trial	Delayed metastasis, improved overall survival in stage II OSA-bearing dogs	Endo-Munoz et al., 2020 [46]
**Branded targets**	**HER-2 CAR-T cells therapy**	OSA tumor initiating cells and human PDX models, in vivo and in vitro Human patients Canine OSA patients	Reduction of tumorigenicity in vitro and reduction of sarcosphere forming efficacy in vivo Tumor and metastasis regression, but severe adverse effects (cytokine storm) Human and canine HER2+ cells killing with no cytokine storm induction	Rainusso et al., 2012 [47]; Morgan et al., 2010 [48]; Mata et al., 2014 [49]
	**Listeria monocytogenes (Lm)-based vaccine expressing a chimeric human HER2 fusion protein**	Phase I dose escalation trial in OSA-bearing dogs Phase Ib clinical trial (ADXS31-164 )	The adjuvant vaccination impaired the development of lung metastasis and in prolonging the overall survival Adult patients with HER2+ tumors, licensed for pediatric OSA patients	Mason et al., 2016 [50]; *Results not published yet*
	**EGFR/HER2 peptide-based vaccination**	Phase I/II veterinary trial in dogs with OSA and other solid tumors	Induction of antibody response and inhibition of the ErbB/HER2 signaling in both canine and human cells in vitro,	Doyle et al., 2021 [51]
	**Anti- IGF-1R monoclonal antibody (Romatumumab)**	Randomized controlled veterinary trial in OSA-bearing dogs Clinical trial in relapsed human OSA patients	No improvement in the survival as compared to chemotherapy alone Few patients displayed complete response, with most of the others developing progressive disease	Khanna et al., 2002 [52]; Anderson et al., 2016 [53]
**Other immunomodulatory strategies**	**Non-conventional ER-stress-response-derived immunogenic peptides (ERstrePs) released upon Salmonella infection**	Veterinary clinical trial including OSA bearing dogs	Development of speific immunity, delayed metastasis and improved survival as compared to historical controls	Marconato et al., 2022 [54]; Melacarne et al., 2021 [55]
	**Liposome-encapsulated lipophilic derivative of muramyl dipeptide (L-MTP-PE )**	Randomized clinical veterinary trial in OSA-bearing dogs Phase I/II clinical trial	Hampered metastatic spread and improved the survival, it was able to enhance both monocyte activation and the cytotoxic activity of macrophages against OSA cells Newly diagnosed or relapsed OSA patients	Macewen et al., 1989 [56]; Kurzman et al., 1995 [57]; Kleinerman et al., 1995 [58]; Shi et al., 1993 [59]; *Ongoing* https://clinicaltrals.gov/ct2/show/NCT04571229, (accessed on 13 December 2022) [60]
	**Losartan + toceranib phosphate (Palladia)**	Veterinary clinical trial in dogs with metastatic OSA	50% of canine patients showed improved survival and achieved stsble disease	Regan et al., 2022 [61]
	**Inhaled rhIL-15 monotherapy**	Phase I veterinary trial in advanced canine OSA patients	Prolonged survival following the induction of a NK-mediated cytotoxic response	Rebhun et al., 2022 [62]

## Data Availability

Not applicable.
